# Achieving complete remission in metastatic hepatocellular carcinoma with sintilimab plus sorafenib therapy followed by hepatic resection: a case report

**DOI:** 10.3389/fonc.2024.1355798

**Published:** 2024-02-06

**Authors:** Kai Cui, Zhongchao Li, Jingtao Zhong, Xuetao Shi, Lei Zhao, Hao Li, Ying Ma

**Affiliations:** ^1^Department of Hepatobiliary Surgery, Shandong Cancer Hospital Affiliated to Shandong First Medical University, Jinan, China; ^2^Shandong Pharmaceutical Research Institute, Shandong First Medical University, Jinan, China

**Keywords:** sintilimab, sorafenib, PD-1 inhibitor, hepatocellular carcinoma, conversion therapy, extrahepatic metastasis

## Abstract

**Background:**

The synergistic effectiveness of combining immune checkpoint inhibitors with targeted therapies has shown promise in improving the conversion rate for unresectable hepatocellular carcinoma (HCC) patients to a potentially resectable status. However, the efficacy of this approach in the context of HCC with extrahepatic metastasis remains to be conclusively determined.

**Case presentation:**

We report a rare case of advanced HCC with extrahepatic metastasis who achieved long-term survival by a combination of systemic therapy (sintilimab and sorafenib) followed by laparoscopic hepatectomy. A 63-year-old man presented at our hospital with discomfort on the right side of his waist. An enlarged right hepatic lobe mass was subsequently revealed by CT scan. The patient’s medical history, including a prior infection with hepatitis B virus, cirrhosis of the liver and an alpha-fetoprotein (AFP) level measuring 41.28 ng/ml substantiated the clinical diagnosis of HCC. On October 30th, 2019, the patient received 200 mg sintilimab intravenously (q3w) plus 200–400 mg BID sorafenib orally, along with antiviral therapy. After six cycles, his disease achieved partial response (PR). On April 26th, 2021, He underwent a laparoscopic hepatectomy. The patient achieved a sustained period of no evidence of disease for 2.5 years and with drug-free survival for 2 years after the resection. His current overall survival is estimated at approximately 4 years.

**Conclusions:**

This case highlights the potential of combining sintilimab and sorafenib in transforming HCC with extrahepatic metastasis into a condition amenable to surgical resection, suggesting that this treatment approach, followed by surgery, may lead to complete remission.

## Background

Hepatocellular carcinoma (HCC), a predominant form of liver cancer, is the third most common cause of cancer-related mortality globally. This cancer type is especially prevalent in China, contributing to almost half of the worldwide cases and deaths (1, 2). Typically diagnosed in its advanced stages, HCC has historically been associated with a median overall survival (OS) of merely nine months for advanced-stage patients (3, 4). Recent developments in treatment, particularly the combination of immune checkpoint inhibitors (ICIs) with anti-angiogenic agents, have emerged as the frontline therapy for advanced HCC. This regime extended the median OS to 20 months (5). The increase in overall response rate (ORR) to 30–40% with this regimen is significant, potentially enabling resection by reducing tumour size or stage, which, in turn, could enhance the conversion rate for HCC treatment.

Here, we present a case of HCC with extrahepatic metastases where a complete remission was achieved through the combination of ICI with an anti-angiogenic agent, followed by a subsequent liver resection, leading to long-term survival.

## Case presentation

In October 2019, a 66-year-old male presented with discomfort on the right side of his waist and was admitted to Jiaozhou Renmin Hospital, China. Initial ultrasound revealed hepatic space-occupying lesions. Subsequent contrast-enhanced computed tomography (CECT) and magnetic resonance imaging (MRI) of the abdomen conducted on 2019-10-22 confirmed multiple lesions in the inferior segment of the right liver lobe. Additionally, the CECT identified cirrhosis of the liver. Laboratory investigations revealed a positive hepatitis B virus (HBV) status with a viral load of 5.10 x 10^3 IU/mL and an alpha-fetoprotein (AFP) level of 41.56 ng/mL. No intervention was initiated.

**Figure 1:**
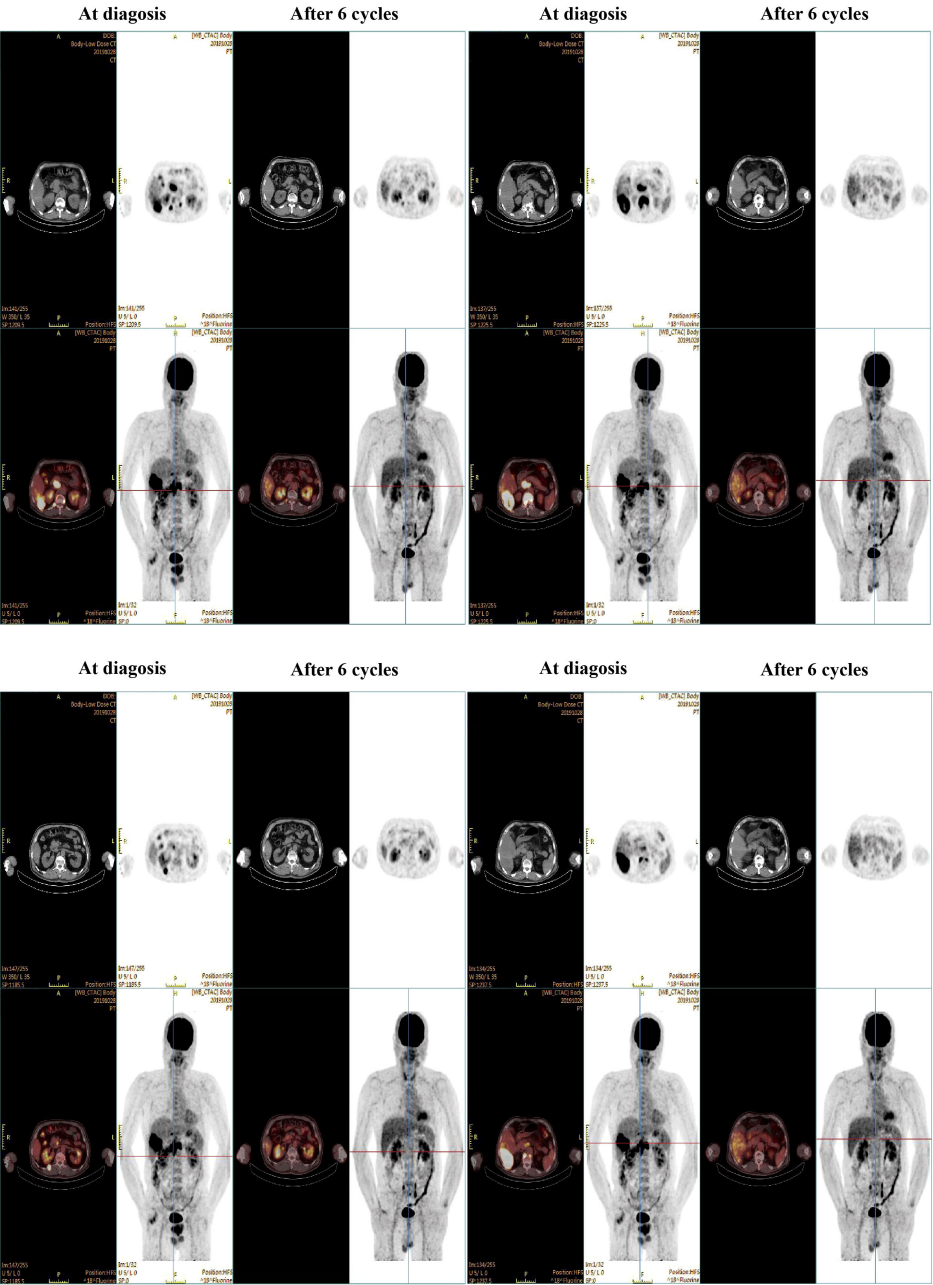
PET-CT scans at diagnosis and after 6 cycles of systemic therapy.

**Figure 2:**
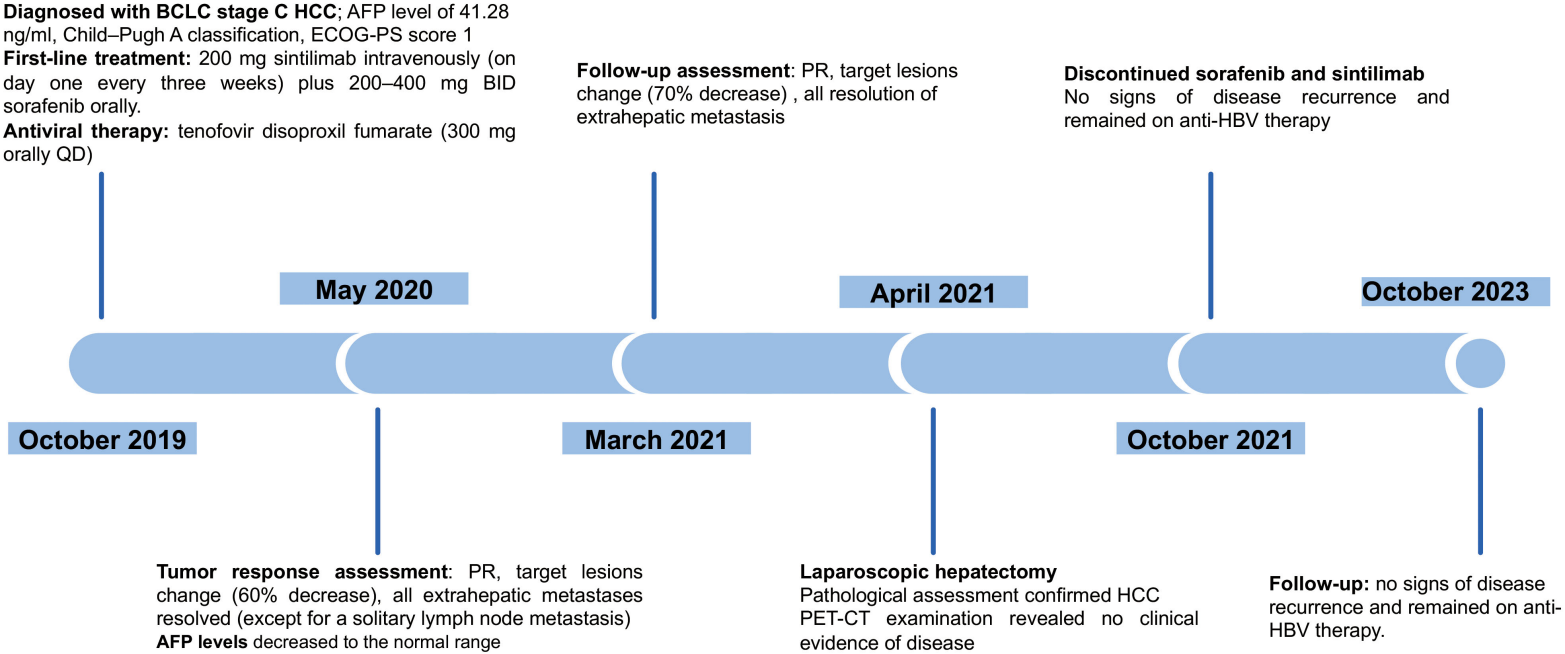
A schematic of course of disease management.

On 2019-10-23, the patient was transferred to our hospital for detailed assessment and subsequent treatment. His medical history of hypertension or diabetes was unremarkable. Physical examination on arrival revealed a flat abdomen with mild right upper quadrant tenderness, although there was no evidence of weight loss or lymphadenopathy. The patient’s Eastern Cooperative Oncology Group (ECOG) score was rated as 1. His liver function was categorised as Child–Pugh class A, with a blood alpha-fetoprotein (AFP) level of 41.28 ng/ml. A positron emission tomography-computed tomography (PET-CT) scan (**Figure 1**) on 2019-10-28 demonstrated an enlarged right hepatic lobe measuring 95 mm×56 mm. Multiple lymph node metastases around the pancreatic head region, posterior to the bilateral diaphragmatic crus and at T10 and T12 on the right side of the vertebral bodies were identified. Additionally, extrahepatic metastatic involvement was noted in the right erector spinae muscle and the T12 vertebral body. The diagnosis was established as Barcelona Clinic Liver Cancer (BCLC) stage C HCC (equivalent to China Liver Cancer [CNLC] stage IIIb) as shown in **Figure 2**.

Given his unresectable tumour, coupled with a Child–Pugh classification of A, a diagnosis of BCLC stage C and an ECOG-PS score of 1, a first-line treatment regimen was initiated for the patient, involving a combination of immunotherapy and targeted therapy. On 2019-10-30, the patient received 200 mg of sintilimab intravenously (on day one every three weeks) plus 200–400 mg BID of sorafenib orally, along with antiviral therapy (tenofovir disoproxil fumarate: 300 mg orally once a day).

**Figure 3:**
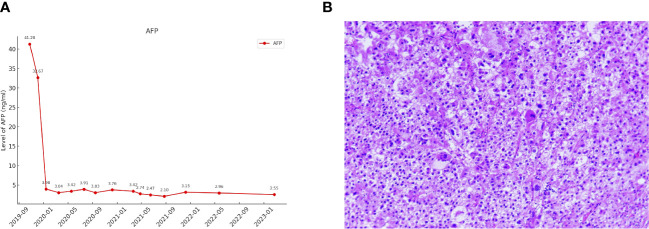
**(A)** The changes in AFP levels during the course of treatment. **(B)** Pathological features of the tumour lesion post-surgery (Hematoxylin and eosin stain, magnification ×200).

After six treatment cycles, an evaluation conducted on 2020-05-21, revealed a significant therapeutic response. His disease achieved PR with a remarkable 60% reduction in the size of the target lesions, measured at 38 mm×23 mm according to RECIST criteria. Moreover, all extrahepatic metastases had been resolved, with the exception of a solitary lymph node metastasis adjacent to the T12 vertebra, measuring ~ 0.8 cm along its short axis with increased glucose metabolism (**Figure 1**). The patient’s AFP levels had decreased substantially from 41.28 ng/ml to within the normal range (as shown in **Figure 3A**), while his liver function remained unimpaired. Furthermore, antiviral therapy had effectively reduced HBV-DNA levels to less than 1.0 x 10^2^ IU/mL, and no adverse events (AEs) were reported during the course of the systemic treatment.

**Figure 4:**
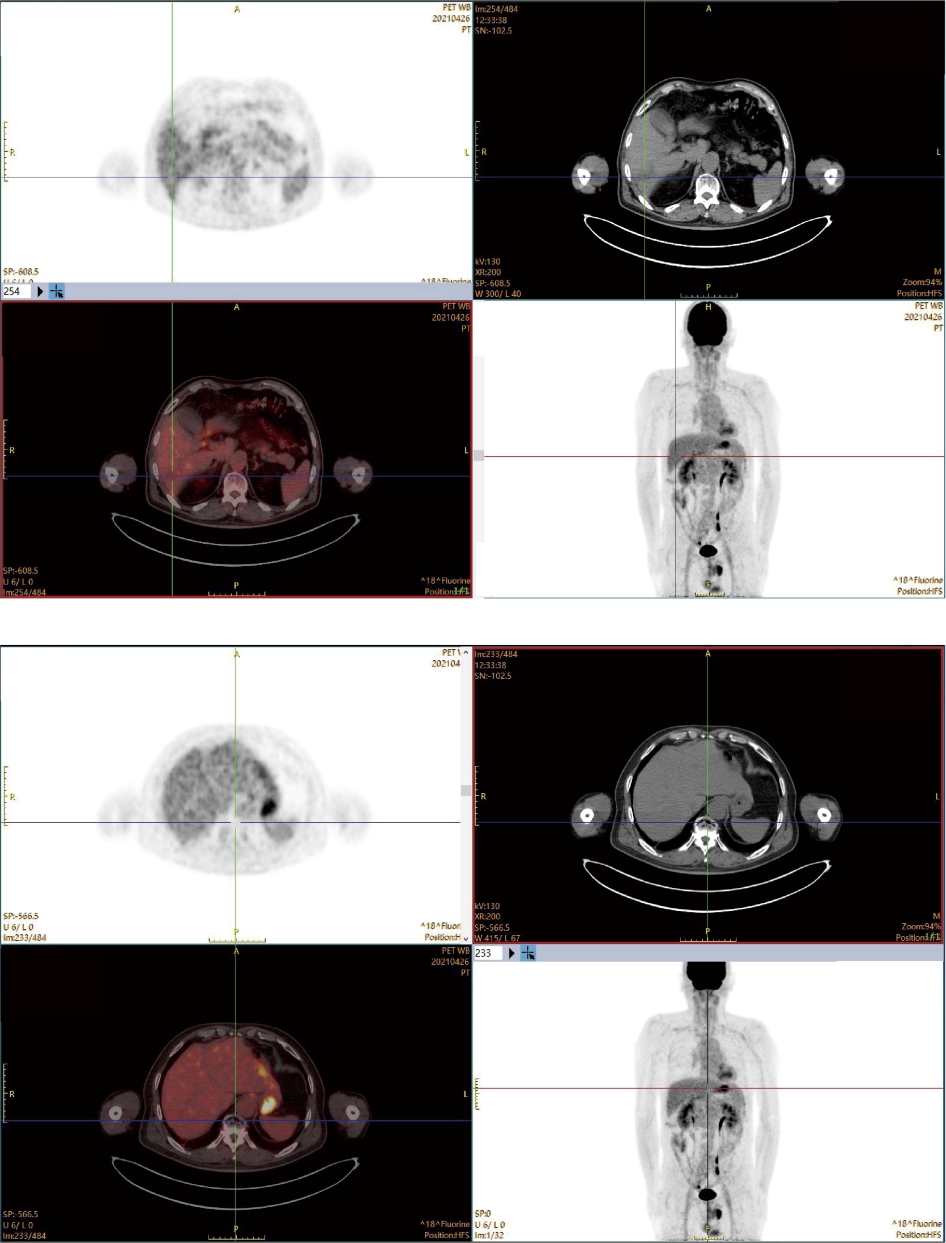
PET images showing no evidence of disease a month after the surgery.

**Figure 5:**
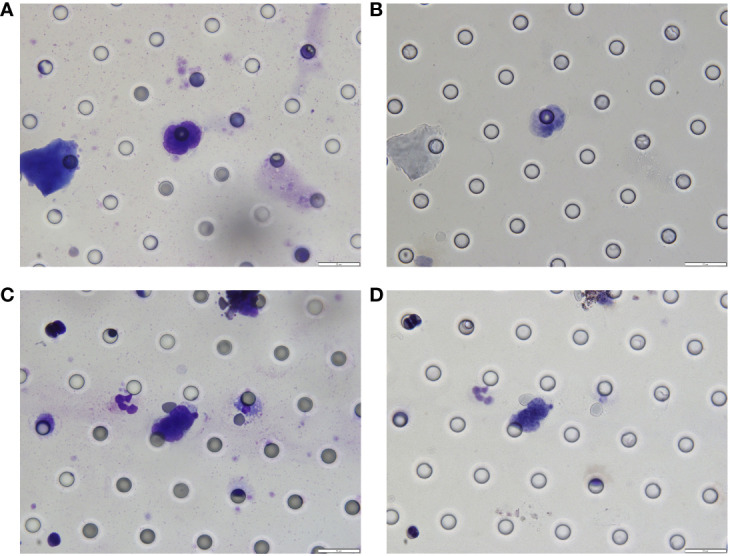
Identification of CTCs by CTCBIOPSY^®^.

The patient continued the therapy for approximately one and a half years. A CT scan conducted on 2021-03-29, revealed a further reduction in the dimensions of liver lesions, now measuring 17 mm×28 mm. This significant response, amounting to a 70% reduction (deep response), along with the resolution of extrahepatic metastasis, prompted the consideration of surgical intervention. On 2021-04-26, a laparoscopic hepatectomy targeting segment 6 (S6) was performed under general anaesthesia. Post-operative pathological assessment confirmed HCC, with post-treatment response evident in the large necrotic area and negative resection margin. A small region of moderately differentiated HCC was encapsulated by fibrous tissue hyperplasia with hyaline degeneration and scattered inflammatory cell infiltration **Figure 3B**). During the post-operative period, no adverse events or complications were observed, and the patient’s liver function remained within normal limits. The patient had an uneventful recovery, without the need for additional clinical intervention. A subsequent follow-up, including a PET-CT examination on 2021-04-26, revealed no clinical evidence of disease (**Figure 4**). Concurrently, analysis via CTCBIOPSY^®^ unveiled a circulating tumour cell count of two per 5 mL of blood, which is considered to be at a level indicating less possibility of disease recurrence (**Figure 5**).

From April 2021 to October 2021, the patient sustained a combined regimen of sorafenib and sintilimab for approximately six months. At the follow-up on October 31, 2023, the patient showed no signs of disease recurrence and remained on anti-HBV therapy.

## Discussion and conclusion

This report describes a case of HCC with extrahepatic metastasis, achieving complete remission with the combination of immunotherapy and anti-angiogenic therapy followed by laparoscopic hepatectomy. Although promising clinical outcomes have been observed in advanced HCC patients without extrahepatic metastases (6–8), patients with BCLC stage C HCC typically rarely receive this treatment modality. The rationale is that widespread extrahepatic metastasis suggests extensive disease spread, making the successful resection of extra-hepatic metastases unlikely in an initial hepatectomy. In this specific case, the patient’s disease was downstaged to an early phase of HCC, rendering the tumour amenable to R0 resection. Subsequent to the discontinuation of systemic therapy, the patient manifested a sustained period of disease-free and drug-free survival, spanning approximately two years. He has persevered for four years following the commencement of the treatment regimen. This case exemplifies the curative potential for this therapeutic strategy in treating advanced HCC, even in cases with extrahepatic metastasis.

Specific predictive biomarkers have been correlated with the efficacy of this treatment strategy. A deep response to the systemic therapy is of potential as one of the indicators suitable for later liver resection with curative intention. For our case, the systemic therapy led to resolved extrahepatic metastasis, with a notable response denoted by a target lesion reduction to 70%. Furthermore, the presence of pre-existing CD8 cells has been recognised as a promising biomarker for gauging the response to treatment with Lenvatinib in combination with anti-PD-1 antibodies (9). Pathological complete response (pCR) has been correlated with favourable outcomes. In our case, the patient did not achieve a pCR according to the hepatological assessments after surgery, thereby suggesting a relatively elevated likelihood of recurrence compared to patients who achieve a pCR. Consequently, a monitoring method – a CTC test – was applied in our case. It has been reported that the mOS was much shorter in the CTC-positive population with HCC, which was also associated with poorer clinical characteristics (10, 11). In our case, the patient’s CTC test results were negative; according to the study by Zhou (12), this indicates a relatively low possibility of disease recurrence.

In addition, for those achieving pCR, the guidance for resection might be informed by CTC or ctDNA-driven minimal residual disease. Over-extensive resection of non-cancerous liver tissue may result in liver dysfunction, accompanied by associated conditions such as ascites, jaundice and hypoalbuminemia. Using CTC or ctDNA, liver resection might be avoided for this population. A recent study reported that among five patients who had achieved a complete remission, three had undergone an R0 resection and have remained free of disease up to the latest follow-up (13). On the other hand, a ‘watch and wait’ approach was chosen by the other two patients. They have sustained a disease-free condition for 7.6 and 19.7 months, respectively. In addition, a current randomised trial in China is assessing the efficacy of surgical resection following systemic treatment with atezolizumab combined with bevacizumab (14). This trial aims not only to reveal the value of hepatectomy in this treatment strategy but also to discern which patients might benefit most from sequential hepatectomy, thereby reducing unnecessary surgeries.

Further investigation for this treatment strategy in advanced HCC is warranted. First, in future studies, it is imperative to ensure a more heterogeneous participant demographic, particularly given that this treatment approach is not commonly applied to BCLC stage C HCC patients. Expanding the cohort to more comprehensively encompass individuals with advanced HCC will significantly bolster the external validity and generalisability of the research outcomes. Second, more evidence is needed to produce a consensus on the optimal timing for resection following successful conversion. According to recent data, within a median of 3.9 months following the onset of systemic therapy, 23.8% (24 out of 101 patients) underwent curative resection, resulting in an improved overall survival for these patients (15). For our case, the patient’s disease achieved PR after six months of the systemic therapy. The interval to liver resection was notably longer: approximately 18 months after initiating systemic treatment. The decision to proceed with the resection earlier might have reduced the time that patients remained on systemic treatment. To effectively navigate the uncertainties surrounding the timing of surgical resection, it is imperative that forthcoming studies meticulously investigate the various factors that sway the clinical decision towards either an immediate or a postponed resection. Third, there is a need for focused research aimed at standardising the length of adjuvant therapy following resection. While comprehensive data are limited, insights from previous studies on adjuvant therapy suggested a postoperative adjuvant treatment duration exceeding six months. Further investigations should consider undertaking controlled trials to evaluate how varying durations of adjuvant therapy influence long-term patient outcomes. Such research could yield more definitive guidelines for clinicians in managing postoperative care. Last but not at least, not all patients may respond optimally to this primary treatment strategy. Therefore, exploring second-line treatment options will be imperative for those who experience disease progression or have suboptimal responses. It has been suggested that regorafenib and ramucirumab notably extend overall survival in comparison to placebos (16). Additionally, cabozantinib, regorafenib, ramucirumab, brivanib, axitinib and pembrolizumab demonstrated a significant enhancement in progression-free survival relative to placebos. However, given the limited efficacy of the current available second line treatments, further clinical trials are still warranted. A phase Ib/II clinical trial has recently shown promising efficacy: the confirmed ORR and disease control rate were 30% (95% CI, 14.6%‐51.9%) from anti‐ALK‐1 monoclonal antibody plus nivolumab and BB as the second line treatment for relapsed advanced HCC patients (17).

In conclusion, this case presents a treatment strategy that successfully achieved complete remission in advanced HCC with extrahepatic metastasis, thereby extending its therapeutic efficacy to the complex cases involving extrahepatic spread.

## Data availability statement

The original contributions presented in the study are included in the article/supplementary material. Further inquiries can be directed to the corresponding author.

## Ethics statement

The studies involving humans were approved by Shandong cancer hospital ethics committee. The studies were conducted in accordance with the local legislation and institutional requirements. The participants provided their written informed consent to participate in this study. Written informed consent was obtained from the individual(s) for the publication of any potentially identifiable images or data included in this article.

## Author contributions

KC: Data curation, Formal analysis, Funding acquisition, Writing – original draft, Writing – review & editing. ZL: Writing – original draft, Writing – review & editing. JZ: Writing – original draft, Writing – review & editing. XS: Writing – original draft, Writing – review & editing. LZ: Writing – original draft, Writing – review & editing. HL: Writing – original draft, Writing – review & editing. YM: Formal analysis, Writing – original draft, Writing – review & editing.
